# A protocol for size separation of nanographenes†

**DOI:** 10.1039/c9ra07528c

**Published:** 2019-10-21

**Authors:** Ikuya Matsumoto, Ryo Sekiya, Takeharu Haino

**Affiliations:** Department of Chemistry, Graduate School of Science, Hiroshima University 1-3-1 Kagamiyama, Higashi-Hiroshima Hiroshima 739-8526 Japan haino@hiroshima-u.ac.jp

## Abstract

Top-down methods are convenient preparative methods for nanographenes, although the products consist of graphene fragments with a broad size distribution. We show that a combination of dialysis membranes (50, 25, 15, 8, and 2 kD) can conveniently separate nanographenes into five size distributions. The separated nanographenes can be employed as starting materials for carbon-based functional materials.

## Abstract 2

Top-down methods are convenient preparative methods for nanographenes, although the products consist of graphene fragments with a broad size distribution. We developed a simple protocol for size separation of nanographenes.

## Graphical abstract

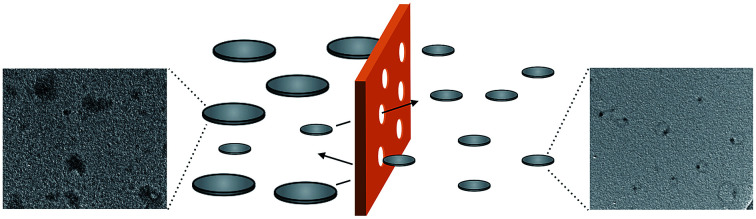

Oxidative cleavage of graphene,^1^ graphene oxide,^2^ graphite,^3,4^ and carbon fibers^5^ by top-down methods produces graphene fragments known as nanographenes, graphene quantum dots, and graphene oxide quantum dots. Nanographenes have band gaps of a few eV due to the quantum size effect,^6^ permitting them to be excited with UV light to emit light in the visible region. Furthermore, they are suggested to have toxicity much lower than that of inorganic quantum dots.^7^ These features make them attractive research targets. Optical materials,^3,4,8–11^ polymers,^12^ bioimaging,^5,13^ photovoltaic devices,^14,15^ electrocatalysts,^16^ and nanomedicines^17^ are examples of their applications.

Top-down methods have several advantages, such as facile production and excellent scalability.^4,5^ From the perspective of product purity, however, top-down methods are inferior to bottom-up methods, which can produce homogeneous nanographenes by organic synthesis.^18^ Uncontrollable oxidative cleavage produces graphene fragments with a broad size distribution. Hence, the properties of nanographenes, such as photophysical properties, are influenced by aspects the production method, such as temperature and reaction time.^5^ To take advantage of top-down methods, the procedures that separate nanographenes into those with narrow size distributions should be developed; such nanographenes are expected to have similar properties.^19^ However, this kind of fundamental study has still been limited, and reported examples rely on chromatographic techniques.^19–23^ Although chromatography is effective for separating nanographenes, expensive instruments, such as preparative-scale HPLC systems, are required for practical use, and not everyone can employ these procedures. Therefore, more convenient, cost-effective, and scalable procedures should be developed.

Herein, we report a gram-scale separation protocol for a nanographene mixture in deionized water. To separate the nanographene mixture, we employed dialysis membranes with five different pore sizes (50, 25, 15, 8, and 2 kD). The fundamental properties of the separated nanographenes are also discussed. This information is valuable for developing carbon-based materials.

Fig. 1 displays a schematic illustration of the separation protocol of nanographenes by dialysis. Twelve grams of graphite purchased from Kanto Chemical Co. was used as the starting material. The oxidative cleavage of graphite in a mixture of sulfuric acid and nitric acid at 120 °C for 24 h followed by neutralization with sodium carbonate and deionization with a dialysis membrane with 2 kD^4^ produced 2.82 g of nanographene mixture. The aqueous solution of the nanographene mixture was stored in a dialysis membrane with a pore size of 50 kD and subjected to dialysis in deionized water. A digital image shows that the water outside the membranes became brown and transparent due to the leak of graphene fragments smaller than the pore sizes. The aqueous solution inside the membranes afforded GQD-1a. The water outside the membrane was concentrated, stored in the dialysis membrane (25 kD) and subjected to dialysis in deionized water. The above procedures were repeated by using dialysis membranes with pore sizes of 15 and 8 kD. The detailed experimental procedures are compiled in ESI.†

**Fig. 1:**
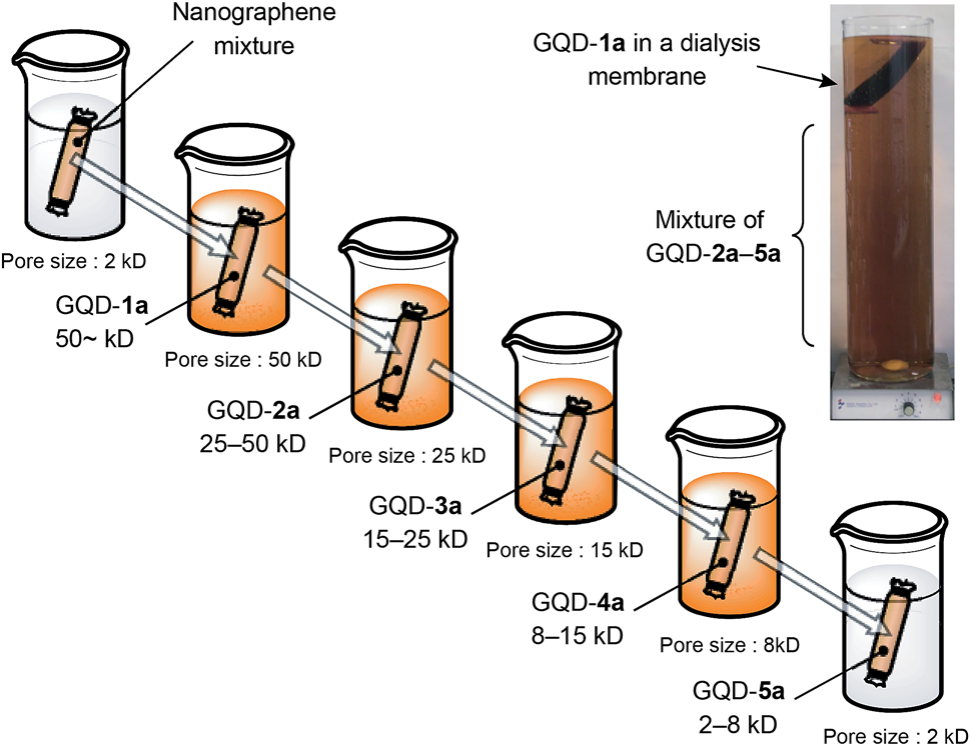
Schematic representation of the separation of nanographene fragments into GQD-1a, GQD-2a, GQD-3a, GQD-4a, and GQD-5a. Inset: a digital image of the dialysis of nanographene mixture by the dialysis membrane of 50 kD.

The aqueous solutions inside the 25, 15, and 8 kD membranes yielded GQD-2a, GQD-3a, and GQD-4a, respectively. The concentration of aqueous solution outside the 8 kD membrane followed by dialysis using the 2 kD membrane to remove the remaining salts gave GQD-5a. The respective fractions were dried *in vacuo* to give brownish-black solids of GQD-1a (348 mg), GQD-2a (301 mg), GQD-3a (318 mg), GQD-4a (406 mg), and GQD-5a (554 mg).^24^

GQD-1a–5a can be directly obtained from the nanographene mixture. For example, 0.343 g of the nanographene mixture was subjected to dialysis using a dialysis membrane (25 kD). The resulting aqueous solution outside the membrane was concentrated and then subjected to dialysis using a dialysis membrane (15 kD) gave 56 mg of nanographenes, which showed a UV-vis spectrum similar to that of GQD-3a (Fig. S13 in ESI†).

The UV-vis spectra of the nanographene mixture and GQD-1a–5a in deionized water showed broad absorption covering most of the visible region (Fig. 2a). The broad absorption originates from the π–π* transitions on the nanographene surface. A shoulder peak in the region of 300–400 nm is assignable to electron transitions from π orbitals of the nanographene to the π* orbital of the carboxy group as well as the n–π* transition within the carboxyl group.^23^ The absorption edge of GQD-1a extended to over 800 nm, and those of the other samples experienced a blueshift, supporting the size separation by the dialysis membranes. The HOMO–LUMO gaps were estimated from the onset of the absorption edge and were in the range of 2.2–2.4 eV.

**Fig. 2:**
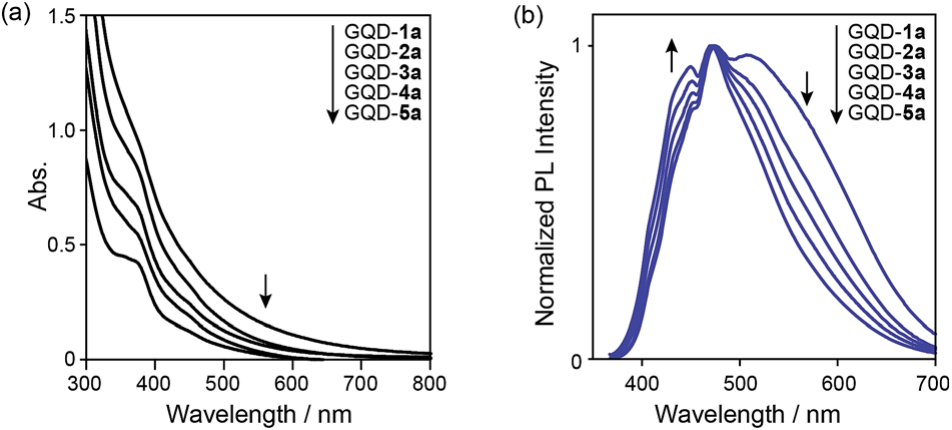
(a) UV-vis absorption spectra of GQD-1a–5a in deionized water. (b) Normalized photoluminescence spectra (*λ*_ex_ = 360 nm) of GQD-1a–5a in deionized water. In both spectra, the concentration of the nanographenes was 0.1 mg mL^−1^.

The photoluminescence (PL) spectra extended throughout the visible region (Fig. S8 in the ESI†). With decreasing nanographene size, the PL in the long-wavelength region (500–700 nm) decreased, while that in the short-wavelength region (400–450) increased (Fig. 2b and S8 in ESI†). The quantum yields (QYs) of GQD-1a–5a (*λ*_ex_ = 500 nm) in water relative to the QY of perylene orange in chloroform^25^ were 0.35%, 0.45%, 0.45%, 0.48%, and 0.48%, respectively, demonstrating that the small graphene fragments are more photoemissive.

To obtain information on the status of the carbon atoms of GQD-1a–5a, X-ray photoelectron spectroscopy (XPS) was performed. The C 1s orbital can be divided into three peaks, of which unoxidized carbons (blue line) were dominant throughout the graphene fragments followed by the C=O (red line) and C–O (green line) bonds (Fig. 3a and S5 in ESI†). The ratio of C=C relative to C=O of GQD-1a was 1 : 0.32, while that of GQD-5a was 1 : 0.52, which reflects the decrease in the nanographene size. ^13^C NMR spectroscopy provided fruitful information on the carbon atoms of GQD-1a–5a (Fig. 3c). Carboxy groups were observed at *δ* = 170–180 ppm. Broad signals assignable to the sp^2^ carbons were observed at *δ* = 135.3, 137.8, and 140.5. Compared to benzene, which shows a signal at *δ* = approximately 128 ppm, these broad signals were found in a more downfield region, likely due to the presence of electron-withdrawing groups near the carbon atoms. Additionally, signals were observed at *δ* = 183.3, 161.0, 129.6 ppm. As the size of the graphene fragments decreased, the signals sharpened, and several signals in the range of 110–125 ppm became recognizable (Fig. 3c-(v)). The DEPT-135 spectrum of GQD-5a demonstrated the observed carbons assignable to quaternary ones (Fig. S4f in ESI†) and little contribution of small aromatic molecules, such as phenol, on the ^13^C NMR spectra.

**Fig. 3:**
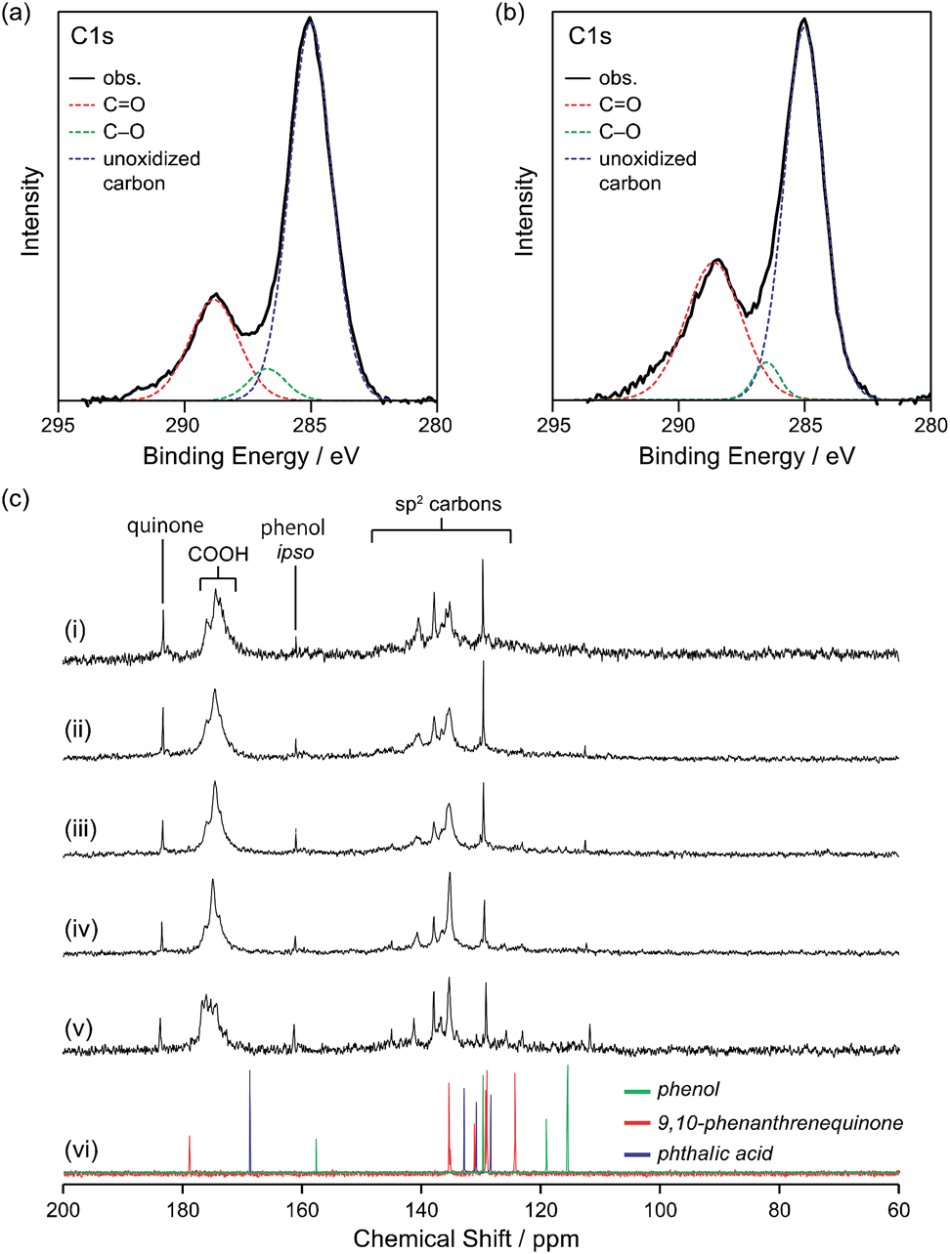
(a) and (b) High-resolution C 1s XPS spectra of (a) GQD-1a and (b) GQD-5a. The C 1s maximum was calibrated to be 285.0 eV. The red, green, and blue dotted lines denote C=O, C–O, and non-oxidized carbon atoms, respectively. (c) ^13^C NMR spectra (75 MHz, 293 K, D_2_O) of (i) GQD-1a, (ii) GQD-2a, (iii) GQD-3a, (iv) GQD-4a, (v) GQD-5a, and (vi) superimposed spectra of phenol, 9,10-phenanthrenequionone, and phthalic acid in DMSO-*d*_6_. Acetone is employed as an internal standard.

To assign the observed signals, ^13^C NMR spectra of phenol, naphthoquinone, 9,10-phenanthrenequinone, and phthalic acid in DMSO-*d*_6_ and phenol in D_2_O were compared with those of GQD-5a (Fig. 3c-(vi), S6, and S7 in ESI†). The signals observed in the high-field region (110–120 ppm) and at *δ* = 161.3 ppm may originate from the *ortho* and *ipso* positions relative to the OH groups on the edge, respectively, and the signal at *δ* = 183.7 ppm is likely to be assigned to quinones on the edge. From the signal intensities, the carboxy group was abundant compared to the other oxygen-containing functional groups.

Polycyclic aromatic hydrocarbons have two distinct edge structures: arm-chair and zig-zag edges. Information on the edge structures is of value for designing carbon-based materials. Because little information regarding the edge structures was available from the IR spectra, edge functionalization of GQD-1a–5a by *p*-methoxybenzyl amine was carried out (Scheme 1). Under the reported reaction conditions,^19^ the edge functionalized nanographenes, GQD-1b–5b, were obtained from GQD-1a–5a. The ATR-IR spectra demonstrated the introduction of the functional groups and the formation of the five-membered cyclic imides at the edge irrespective of their size,^26,27^ indicative of the arm-chair edge with two carboxy groups on the edge as dominant edge structures of GQD-1a–5a (Fig. 4a and S10†). These observations suggested that the arm-chair edges with two carboxylic groups are generated in every stage of the oxidative cleavage (Fig. 4b). The arm-chair edge is more stable than the zig-zag edge.^28–31^ Hence, the latter edges may be decomposed during the oxidative cutting, and as a result, the former edges remained in GQD-1a–5a.

**Scheme 1:**
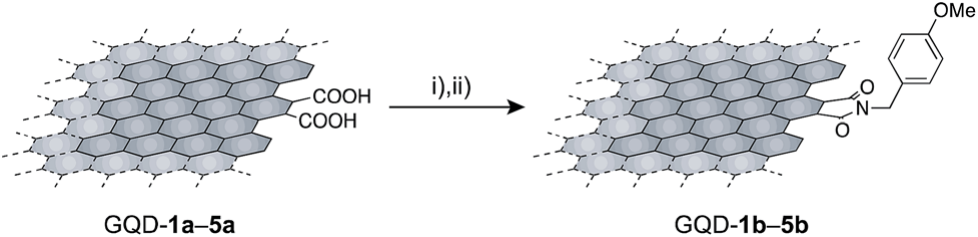
Edge functionalization of GQD-1a–5a by *p*-methoxybenzyl amine. Reaction conditions: (i) (COCl)_2_, 60 °C, 4 days; (ii) DMF + Et_3_N (1 : 1, v/v), *p*-methoxybenzylamine, 80 °C, 2 days.

**Fig. 4:**
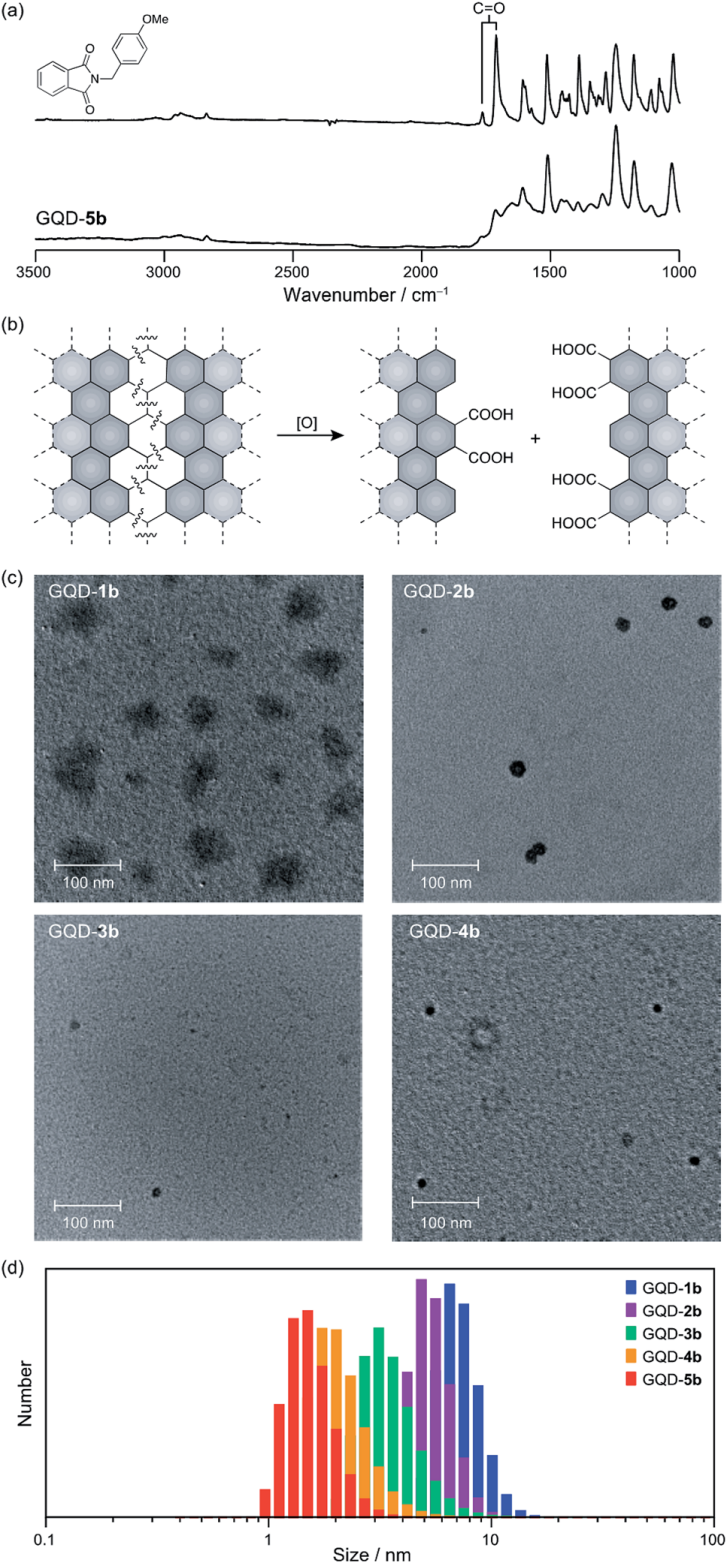
(a) Selected region of ATR-IR spectra of (top) a model compound and (bottom) GQD-5b. (b) A proposed oxidative cleavage on a carbon sheet. (c) Transmission electron microscopy images of GQD-1b–4b. The concentrations of GQD-1b–4b are 0.1 mg mL^−1^. (d) Dynamic light scattering analysis (293 K, CH_2_Cl_2_) of GQD-1b (blue), GQD-2b (purple), GQD-3b (green), GQD-4b (orange), and GQD-5b (red). The concentration of the functionalized nanographenes is 2.5 × 10^−2^ mg mL^−1^.

The improved solubility for organic solvents led us to carry out transmission electron microscopy (TEM) measurements of GQD-1b–4b (Fig. 4c). Large nanographene fragments with a broad size distribution (40–70 nm) were observed in the TEM image of GQD-1b, while only small fragments (15–20 nm) were observed in the TEM image of GQD-4b.

The dynamic light scattering (DLS) analysis of GQD-1b–5b in dichloromethane supported the size separation (Fig. 4d). The size distributions were GQD-1b (7.3 ± 0.2 nm), GQD-2b (5.3 ± 0.1 nm), GQD-3b (3.58 ± 0.02 nm), GQD-4b (2.15 ± 0.07 nm), and GQD-5b (1.56 ± 0.04 nm). The order of the size distribution was consistent with the pore size of the dialysis membranes used in the separation experiments, although the figures provided by the DLS analysis underestimated the size of the nanographenes.

Edge functionalization changed the photophysical properties (Fig. S11 in ESI†), including the QYs. The QYs of GQD-1b–5b in dichloromethane solutions relative to perylene orange were 1.1%, 1.2%, 1.0%, 1.1%, and 1.7%, respectively, demonstrating that the chemical functionalization enhances the QY of the nanographene fragments. Again, the smallest nanographene displayed the highest QYs. We speculate that the organic functional groups suppress the aggregation of the nanographenes, likely due to steric contacts between the functional groups, preventing aggregation-induced quenching.

## Conclusions

In conclusion, a convenient, cost-effective, gram-scale separation protocol for graphene fragments in deionized water was developed. By employing dialysis membranes with five different pore sizes, the graphene fragments were divided into those with five size distributions. This protocol permits the separation of specific nanographenes from the mixture by combining two dialysis membranes. Although the chemical structures of the respective fragments were similar to each other, small graphene fragments are more photoemissive than larger ones. As demonstrated by the edge modification with the *p*-methoxybenzyl group, the respective graphene fragments can be employed as starting materials for carbon-based functional materials.

## Conflicts of interest

There are no conflicts to declare.

## Supplementary Material

RA-009-C9RA07528C-s001
