# Supplementary material for: A protocol for size separation of nanographenes
Source: RSC Adv. 2019 Oct 21;9(58):33843–6. doi: 10.1039/c9ra07528c (PMC9073626; doi:10.1039/c9ra07528c)
Supplement: RA-009-C9RA07528C-s001 [file RA-009-C9RA07528C-s001.pdf]

Electronic Supplementary Material (ESI) for RSC Advances.
This journal is © The Royal Society of Chemistry 2019

Electronic Supplementary Information

A Protocol for size separation of nanographenes

Ikuya Matsumoto, Ryo Sekiya, and Takeharu Haino*

[1] Department of Chemistry, Graduate School of Science, Hiroshima University, 1-3-1 Kagamiyama, Higashi-Hiroshima, Hiroshima 739-8526, Japan

To whom correspondence should be addressed

haino@hiroshima-u.ac.jp

Contents

Experimental Section

• General ··· S3
• Dynamic light scattering analysis ··· S3
• Transmission electron microscope measurements ··· S3
• X-ray photoelectron spectroscopy ··· S3
• **Scheme S1.** Schematic representation of the separation procedure of the nanographene mixture ··· S4
• **Figure S1.** Digital image of dialysis membranes in deionized water ··· S4
• Separation of the nanographene mixture by dialysis membranes ··· S5–S6
• Synthesis of GQD-**1b**–**5b** ··· S6

Supporting Figures and Table

• **Figure S2.** ATR-IR spectra of GQD-**1a**–**5a** ··· S7
• **Figure S3.** ^{1}H NMR spectra of GQD-**1a**–**5a** in D_2O ··· S7
• **Figure S4.** ^{13}C NMR spectra of GQD-**1a**–**5a** and DEPT spectrum of GQD-**5a** in D_2O ··· S8
• **Figure S5.** XPS spectra of the C 1s orbital of GQD-**2a**, GQD-**3a**, and GQD-**5a** ··· S8
• **Figure S6.** Comparison of ^{13}C NMR spectrum of GQD-**5a** with those of four organic compounds ··· S9
• **Figure S7.** Comparison of ^{13}C NMR spectrum of GQD-**5a** with that of phenol in D_2O ··· S9
• **Figure S8.** UV-vis and photoluminescence spectra of GQD-**1a**–**5a** in deionized water ··· S10
• **Figure S9.** Normalized photoemission spectra of GQD-**1a**–**5a** in D_2O ··· S11
• **Figure S10.** ATR-IR spectra of GQD-**1b**–**5b** and a model compound ··· S12
• **Figure S11.** UV-vis and photoluminescence spectra of GQD-**1b**–**5b** in dichloromethane ··· S13
• **Figure S12.** Optimized structure of the model compound ··· S14
• **Figure S13.** UV-vis absorption spectra of GQD-**3a** and GQD-**3a** collected from the mixture ··· S14
• **Table S1.** Cartesian coordinates of the optimized structure of the model compound ··· S15–16

References

• **References** ··· S17

Experimental Section

General

All chemicals and solvents were purchased from Kanto Chemical Co., Ltd., Wako Pure Chemical Co., Ltd., Tokyo Kasei Kogyo Co., Ltd., and Sigma-Aldrich Co., Ltd., and were used as received without further purification. ^{1}H and ^{13}C NMR spectra were recorded on a VARIAN 300 MHz spectrometer. Chemical shifts are quoted as parts per million (ppm) relative to acetone dissolved in D_2O (δ = 2.22 ppm for ^{1}H and 30.9 ppm for ^{13}C) or DMSO (δ = 39.5 ppm for ^{13}C). IR spectra were recorded on a JASCO FT/IR-4600 spectrometer with ZeSe ATR accessory. The synthesis of the nanographene mixture and a model compound were reported previously.[1]

Dynamic Light-Scattering Analysis

Dynamic light-scattering (DLS) analysis was carried out on a Malvern zetasizer nanoZS. The particle size distributions of GQD-**1b–5b** in dichloromethane were measured at 25 °C with a detection angle of 90°. The concentrations of the solutions were 0.025 mg mL^{-1}.

Transmission Electron Microscope Measurements

Transmission electron microscope (TEM) images of GQD-**1b** and GQD-**4b** were obtained on a JEM-2010 system. Samples for TEM imaging were prepared by drop casting of dispersions onto Cu plates, which were then allowed to dry in under reduced pressure. The solution prepared were approximately 0.1 mg mL^{-1}.

X-ray Photoelectron Spectroscopy

Survey scan and high resolution XPS spectra of GQD-**1a**–**5a** were recorded on a Shimazu ESCA-3400 electron spectrometer with Mg Kα radiation. Samples for XPS measurements were prepared by drop casting of the deionized aqueous solutions of the samples onto glass plates, which were then allowed to dry under reduced pressure.

Separation of the nanographene mixture by the dialysis membrane

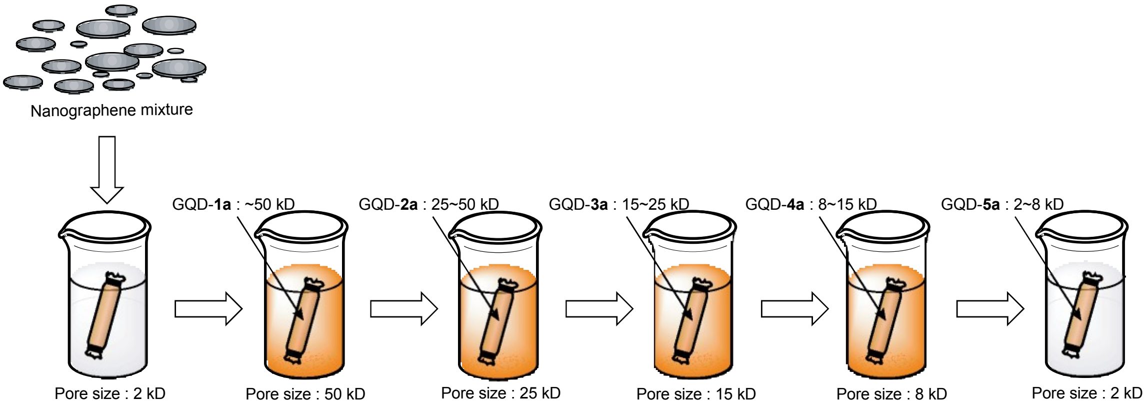

Scheme S1. Schematic representation of the separation of the nanographene mixture by the dialysis membranes with five different pore sizes.

Nanographenes were obtained by the reaction reported previously.[1] In each experiment, 2.0 g of graphite was used. The reactions were conducted by six times (total amount of graphite used was 12.0 g). As prepared nanographene was neutralized with Na_2CO_3 and precipitated inorganic salts (Na_2SO_4 and $NaNO_3$) were filtered off. The black aqueous solution was concentrated and subjected to deionization with Spectra/Por7®Dialysis Membrane with pore size of 2 kD in deionized water. After the deionization, the solution in the dialysis membrane was acidified with aqueous hydrogen chloride to pH~3 and then the acidified aqueous solution was deionized again with the same dialysis membrane.

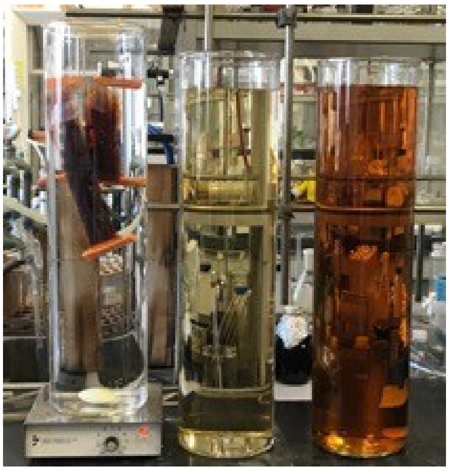

Figure S1. Digital image of the first (right), (b) second (middle), and (c) third (left) dialysis. The nanographenes smaller than the pore size of the given dialysis membrane leaked off from the membranes, while the larger ones remained in the membranes. The deionized waters became brown in the first dialysis, yellow in the second dialysis, and colorless in the third dialysis, indicating that the separation was finished. The three deionized waters (ca. 6 L each) were combined and concentrated. The resultant black aqueous solution was used in the next dialysis.

Dialysis membrane (50 kD)

The solution in the membrane was moved to Spectra/Por7® Dialysis Membrane with the pore size of 50 kD and subjected to dialysis in deionized water (ca. 6 L). The dialysis was carried out by three times for 8 hours (first and second dialysis) or 4 hours (third dialysis) at room temperature. As shown in Figure S1, in the first dialysis, the aqueous solution outside the membrane became brown due to the leaking off nanographenes smaller than the diameter of the pores from the membrane. In the second dialysis, the aqueous solution outside the membrane became brownish yellow and in the third dialysis, the aqueous solution became pale-yellow. The three aqueous solutions (ca. 18 L) were combined and concentrated. The resulting aqueous solution was used in the next dialysis (dialysis membrane with the pore size of 25 kD). The aqueous solution remained in the membranes were concentrated under reduced pressure and the resultant black solid was dried in vacuo for a couple of hours to give GQD-**1a**.

Dialysis membrane (25 kD)

Spectra/Por7® Dialysis Membrane with the pore size of 25 kD was used. The concentrated aqueous solution was moved to this membrane and was subjected to dialysis in deionized water (ca. 6 L). The dialysis was carried out by three times at room temperature. The three aqueous solutions (ca. 18 L) were combined and concentrated. The resulting concentrated aqueous solution was used in the next dialysis (dialysis membrane with the pore size of 15 kD). The aqueous solution remained in the membranes were concentrated under reduced pressure and the resultant black solid was dried in vacuo for a couple of hours to give GQD-**2a**.

Dialysis membrane (15 kD)

Spectra/Por7® Dialysis Membrane with the pore size of 15 kD was used. The concentrated aqueous solution was moved to this membrane and was subjected to dialysis in deionized water (ca. 6 L). The dialysis was carried out by three times at room temperature. The three aqueous solutions (ca. 18 L) were combined and concentrated. The resulting concentrated aqueous solution was used in the next dialysis (dialysis membrane with the pore size of 8 kD). The aqueous solution remained in the membranes were concentrated under reduced pressure and the resultant black solid was dried in vacuo for a couple of hours to give GQD-**3a**.

Dialysis membrane (8 kD and 2 kD)

Spectra/Por7® Dialysis Membrane with the pore size of 8 kD was employed. The concentrated aqueous solution was moved to this membrane and was subjected to dialysis in deionized water (ca. 6 L). The dialysis was carried out by three times at room temperature. The aqueous solution remained in the membranes were concentrated under reduced pressure and the resultant black solid was dried in vacuo for a couple of hours to give GQD-**4a**. The three aqueous solutions (ca. 18 L) were combined and concentrated to give black solid. Since very small amount of inorganic salts remains in deionized water, the repeated concentration accumulated the inorganic slats. To remove the salt completely

from the black solid, it was subjected to dialysis with Spectra/Por7® Dialysis Membrane with the pore size of 2 kD. The aqueous solution inside the membrane was removed under reduced pressure and dried in vacuo to give GQD-**5a**.

Characterization of GQD-1a–5a

GQD-**1a**–**5a** were characterized by ^{1}H and ^{13}C NMR (Figures S3, S4, S6, and S7), IR (Figure S2), and XPS (Figures 4a and S5) spectra. The signals assignable to the sp^2 carbons of the nanographene sheets (δ = 120–140 ppm) and the sp^2 carbons of the carboxyl groups on the edges (δ = 170–180 ppm) were observed in the ^{13}C NMR spectra. In addition to these signals, several signals were found in the aromatic region. The ^{1}H NMR spectra show two weak signals between 7.5 and 8.5 ppm, both of which might come from the nanographene edge.

Synthesis of GQD-1b–5b

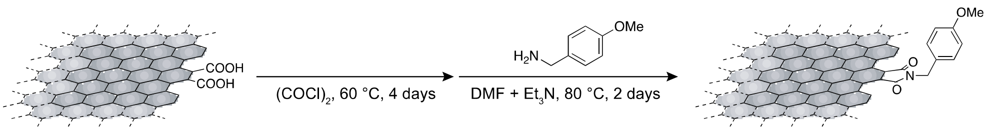

Edge-functionalization by *p*-methoxybenzylamine was reported previously.[2] To a solution of GQD-**5a** (20 mg) in oxalyl chloride (5 mL) was added dry *N*,*N*-dimethylformaide (20 μL). The mixture was subjected to ultrasonic treatment for 3 h and stirred for 4 days at 60 °C. The mixture was cooled to room temperature, and oxalyl chloride was removed in vacuo. The residue was dissolved in dry *N*,*N*-dimethylformamide (5 mL), and *p*-methoxybenzylamine (5.0 mL, 38 mmol) and triethylamine (120 μL) were added to the solution. The mixture was stirred for 2 days at 80 °C. It was then cooled to room temperature, and the solution was poured into water. Precipitate was obtained by filtration and was dissolved in dichlromethane. The solution was washed with deionized water and concentrated in vacuo. Purification of the reaction mixture by column chromatography on BioBeads S-X1 (THF) afforded GQD-**5b** (38.4 mg) as a brown solid.

GQD-**1b**–**4b** were synthesized by the similar methods using GQD-**1a**–**4a** as starting materials. The characterization of the *p*-methoxybenzyl group installed nanographenes were reported in Ref. 2.

Supporting Figures

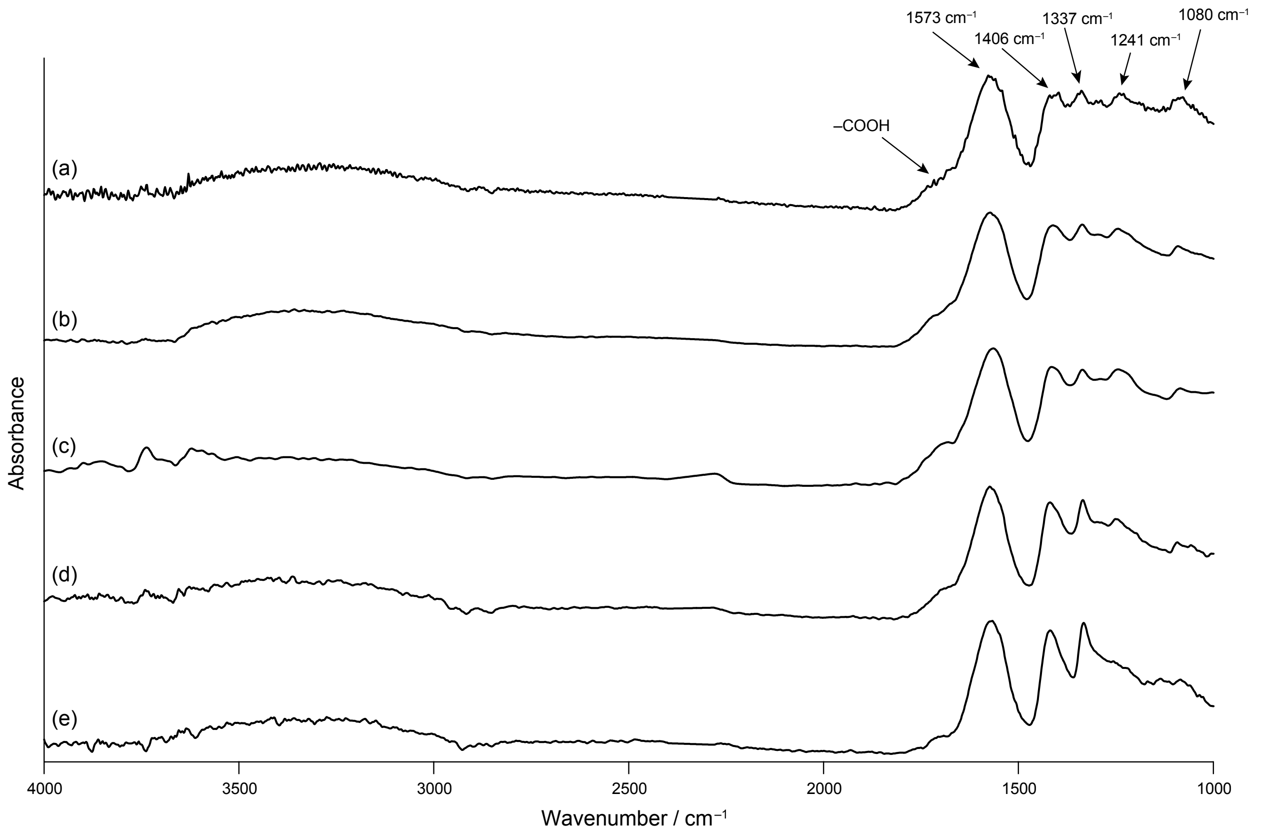

Figure S2. ATR-IR spectra of (a) GQD-**1a**, (b) GQD-**2a**, (c) GQD-**3a**, (d) GQD-**4a**, and (e) GQD-**5a**.

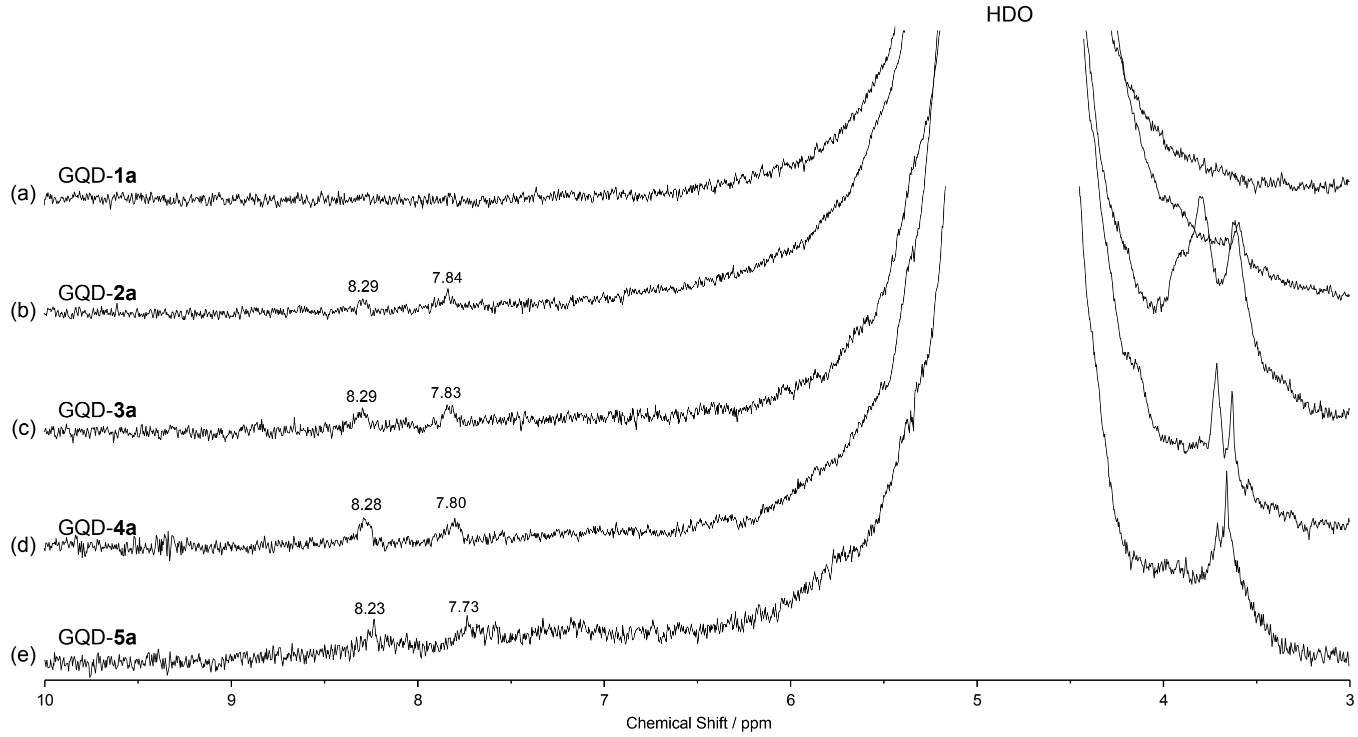

Figure S3. ^{1}H NMR spectra (300 MHz, D_2O, 293 K) of (a) GQD-**1a**, (b) GQD-**2a**, (c) GQD-**3a**, (d) GQD-**4a**, and (e) GQD-**5a**.

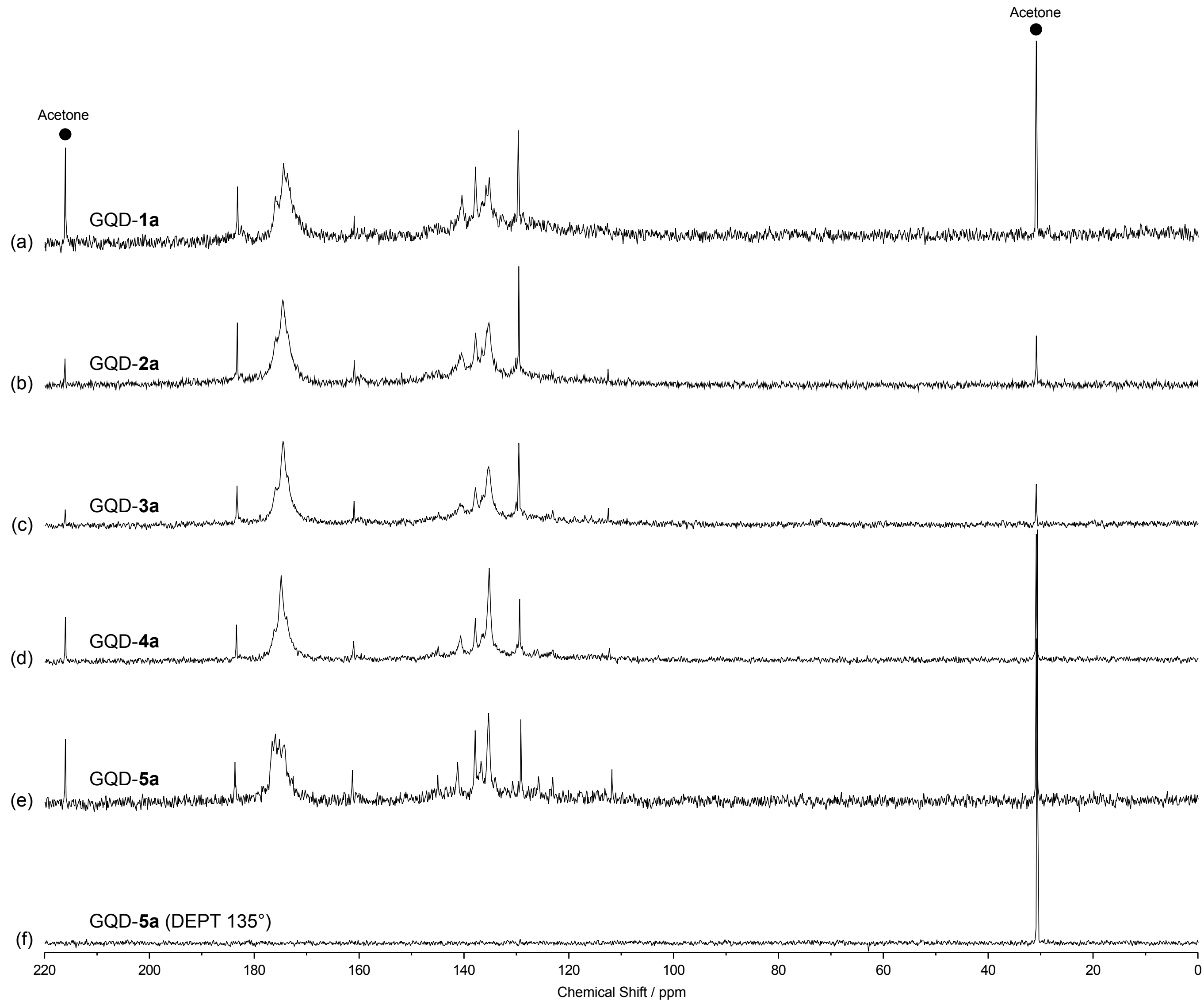

Figure S4. ^{13}C NMR spectra (75 MHz, D_2O, 293 K) of (a) GQD-**1a**, (b) GQD-**2a**, (c) GQD-**3a**, (d) GQD-**4a**, and (e) GQD-**5a**. (f) DEPT-135 spectrum (75 MHz, D_2O, 293 K) of GQD-**5a**. (a)–(f) Acetone was used as an internal standard.

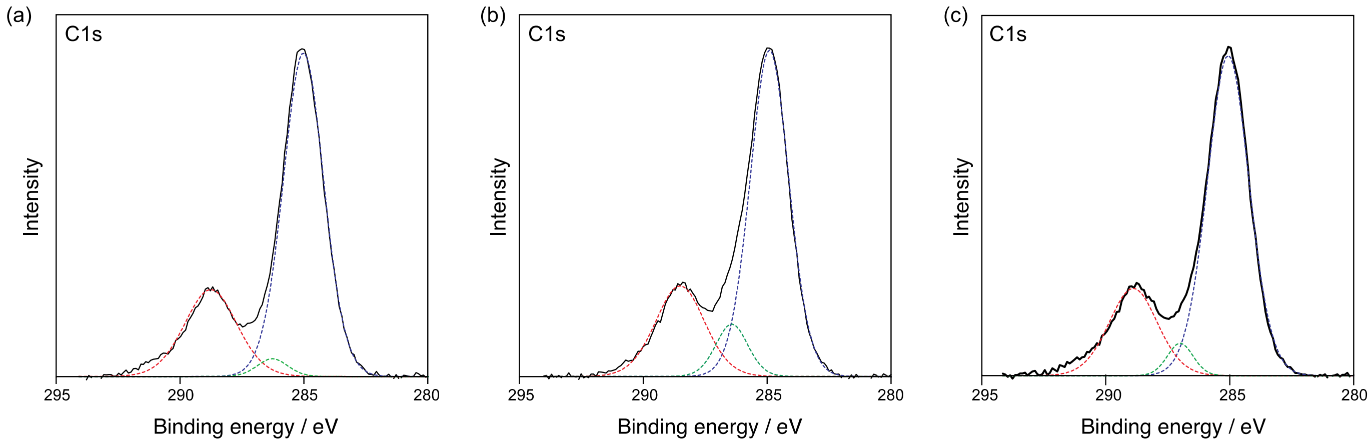

Figure S5. High resolution XPS spectra of the C1s orbital of (a) GQD-**2a**, (b) GQD-**3a**, and (c) GQD-**4a**. The peak maximum was calibrated to be 285.0 eV. The red, green, and blue dotted lines denote C=O, C–O, and non-oxidized carbon atoms, respectively.

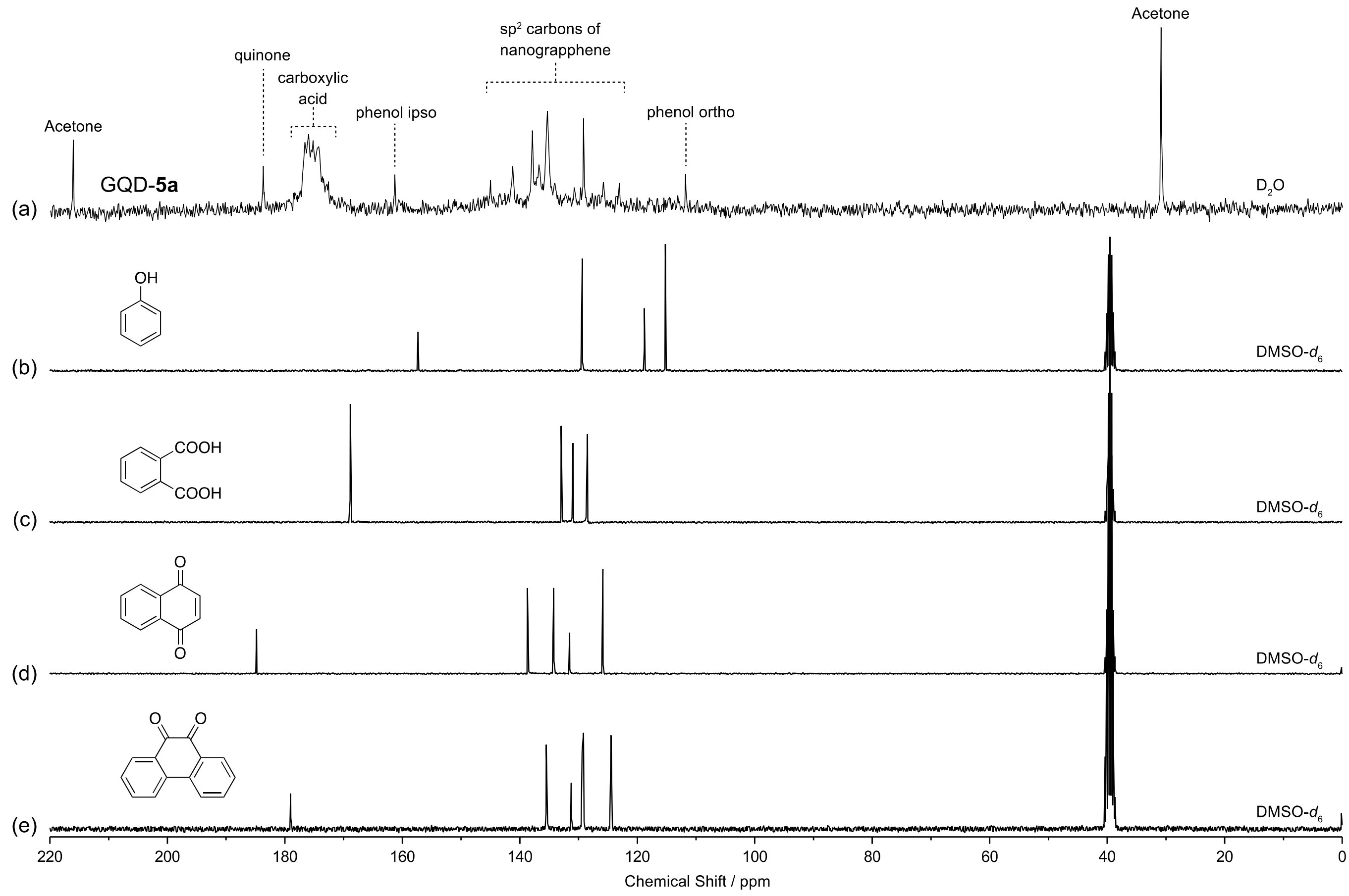

Figure S6. Comparison of ^{13}C NMR spectrum (75 MHz, 293 K) of (a) GQD-**5a** in D_2O with those of (b) phenol, (c) phthalic acid, (d) naphthoquinone, and (e) 9,10-phenanthrenequinone in DMSO-d_6.

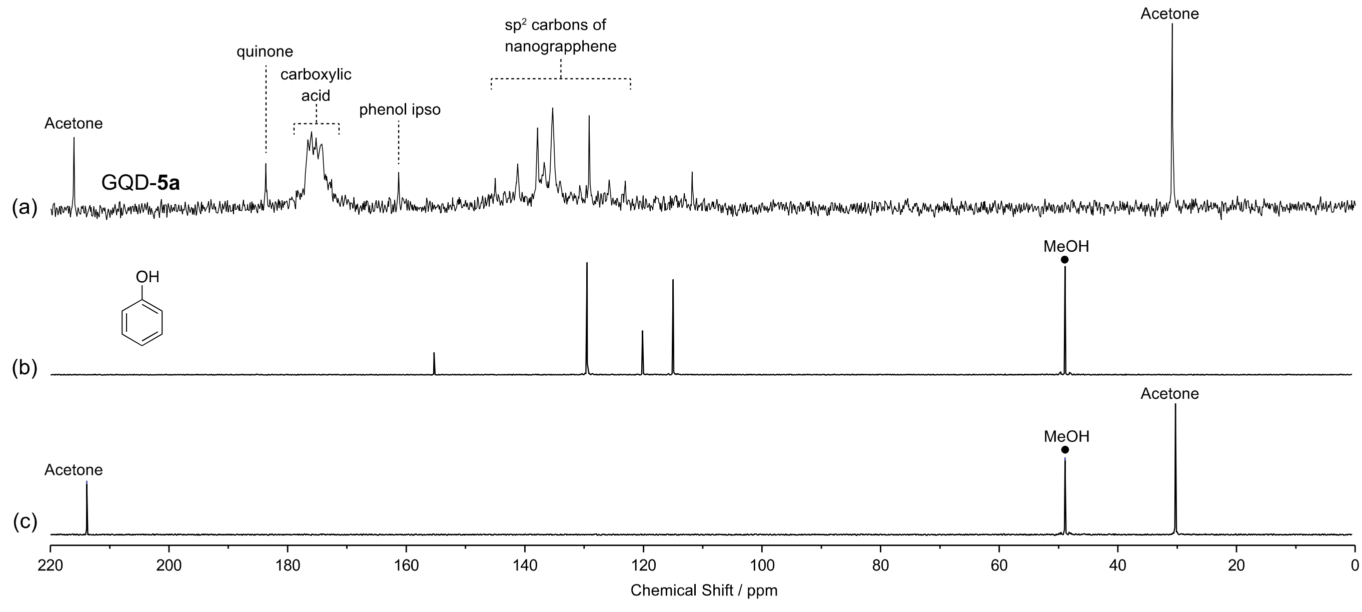

Figure S7. Comparison of ^{13}C NMR spectrum (75 MHz, D_2O, 293 K) of GQD-**5a** with that of phenol. The chemical shift of phenol in D_2O was relative to that of methanol. The chemical shift of methanol in D_2O was relative to that of acetone.

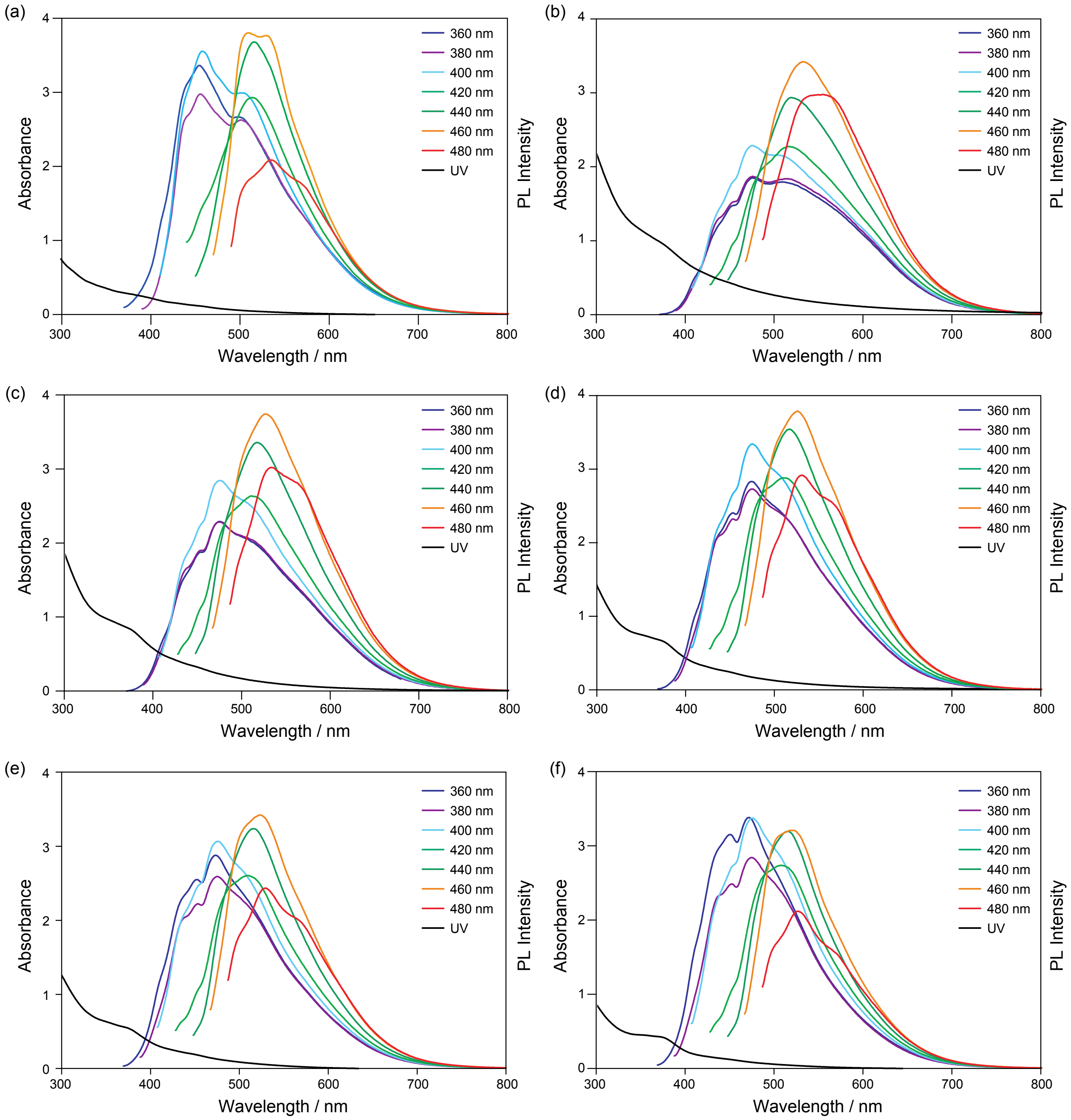

Figure S8. UV-vis and photoemission spectra (H_2O, 298 K) of (a) the nanographene mixture (before separation), (b) GQD-**1a**, (c) GQD-**2a**, (d) GQD-**3a**, (e) GQD-**4a**, and (f) GQD-**5**a Excitation wavelength (λ_{ex}) = 360, 380, 400, 420, 440, 460, and 480 nm. Concentration of the aqueous solutions are 0.1 mg mL^{-1}.

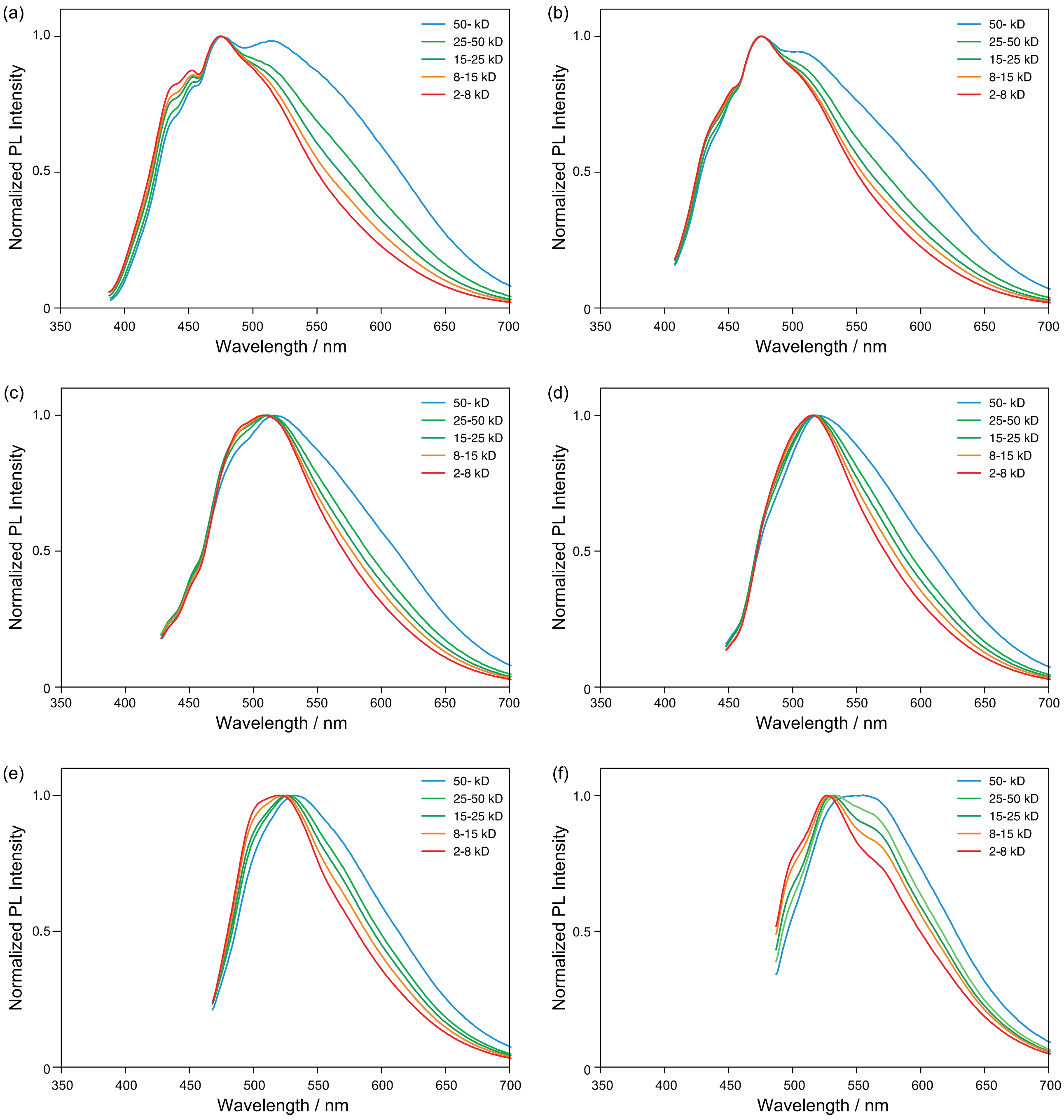

Figure S9. (a)–(f) Normalized photoemission spectra (H_2O, 298 K) of GQD-**1a**–**5a**. (a) λ_{ex} = 380 nm, (b) λ_{ex} = 400 nm, (c) λ_{ex} = 420 nm, (d) λ_{ex} = 440 nm, (e) λ_{ex} = 460 nm, and (f) λ_{ex} = 480 nm. Concentration of the aqueous solutions are 0.1 mg mL^{-1}

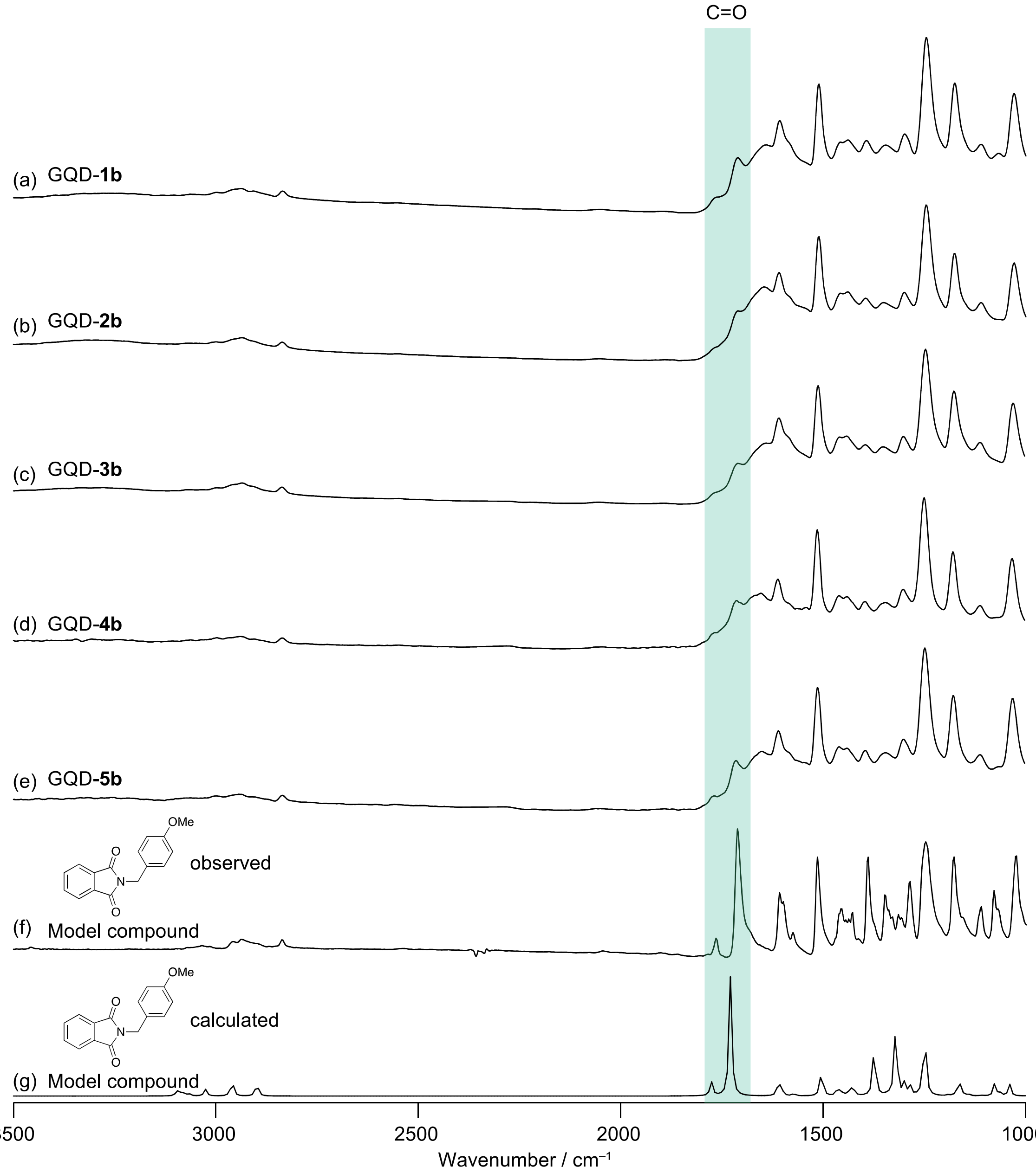

Figure S10. Observed and calculated IR spectra of (a) GQD-**1b**, (b) GQD-**2b**, (c) GQD-**3b**, (d) GQD-**4b**, (e) GQD-**5b**, (f) model compound, and (g) model compound calculated by the *Gaussian 09* program using B3LYP/6-31G(d,p) level of theory. The synthesis of the model compound can be found in Ref. 2.

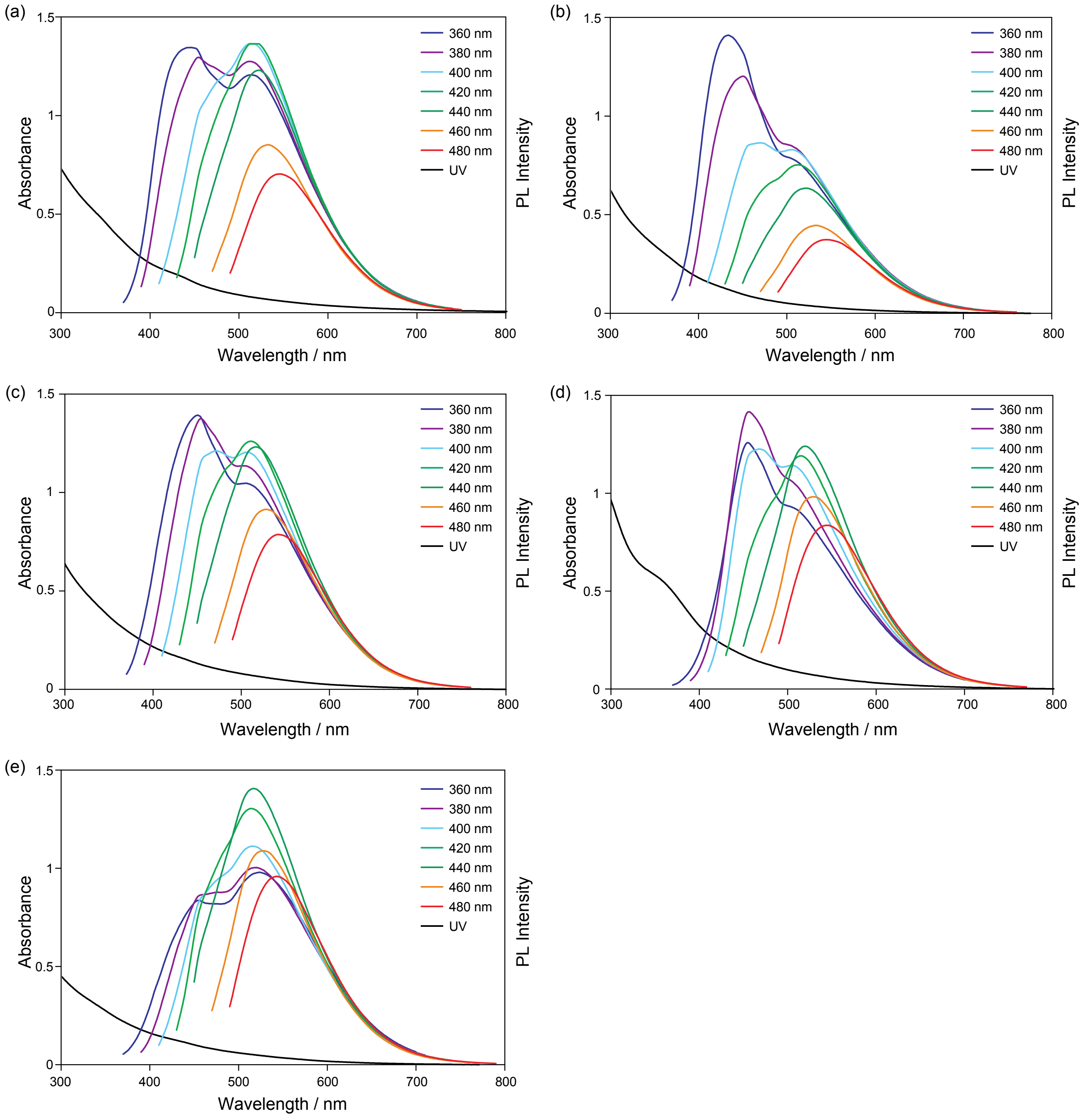

Figure S11. UV-vis absorption spectra (dichloromethane, 298 K) of (a) GQD-**1b**, (b) GQD-**2b**, (c) GQD-**3b**, (d) GQD-**4b**, and (e) GQD-**5b**.

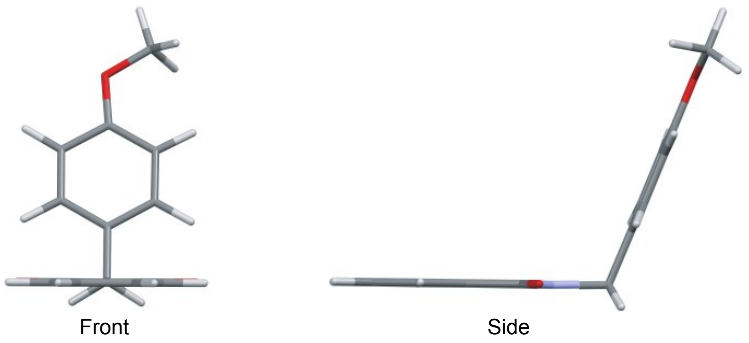

Figure S12. An energy minimized structure of model compound by the *Gaussian 09* program using B3LYP/6-31G(d,p) level of theory.

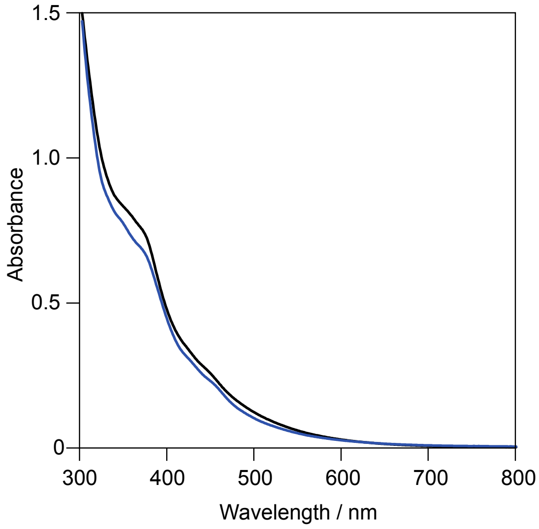

Figure S13. UV-vis absorption spectra (deionized water, 298 K) of GQD-**3a** and GQD-**3a** directly collected from the nanographene mixture.

Table S1. Cartesian coordinate of model compound.

Center Number	Atomic Number	Atomic Type	Coordinates (Angstroms) X	Y	Z
1	6	0	2.751618	-0.762989	0.057326
2	6	0	1.563348	-0.952875	0.943256
3	6	0	3.688713	-1.680032	-0.394117
4	6	0	2.818227	0.585406	-0.300240
5	6	0	3.824089	1.068712	-1.123199
6	6	0	4.708732	-1.202154	-1.227576
7	6	0	4.775262	0.149791	-1.586290
8	6	0	1.674902	1.297310	0.346982
9	1	0	3.865733	2.118509	-1.394333
10	1	0	3.627288	-2.725250	-0.109302
11	1	0	5.460711	-1.889777	-1.602585
12	1	0	5.577749	0.489439	-2.234022
13	8	0	1.154952	-1.970359	1.469045
14	8	0	1.375079	2.474255	0.290594
15	7	0	0.982846	0.317132	1.073284
16	6	0	-0.239831	0.579157	1.837619
17	1	0	-0.152788	1.599192	2.219186
18	6	0	-1.507288	0.417864	1.024715
19	1	0	-0.231840	-0.114185	2.681806
20	6	0	-2.164898	-0.812280	0.961783
21	6	0	-2.036595	1.497570	0.300586
22	6	0	-3.190958	1.350789	-0.456296
23	6	0	-3.327652	-0.976441	0.204763
24	6	0	-3.845965	0.111028	-0.507688
25	1	0	-1.528537	2.457451	0.327002
26	1	0	-3.608605	2.180752	-1.016608
27	1	0	-1.759129	-1.661800	1.504036
28	1	0	-3.813883	-1.944244	0.180557
29	8	0	-4.974186	0.070298	-1.275637
30	6	0	-5.679532	-1.157113	-1.368628
31	1	0	-5.059695	-1.949267	-1.807821
32	1	0	-6.044706	-1.490887	-0.388858
33	1	0	-6.532003	-0.965886	-2.022046

References

[1] R. Sekiya, Y. Uemura, H. Murakami, T. Haino, *Angew. Chem. Int. Ed.,* **2014**, *53*, 5619–5623.

[2] K. Yamato, R. Sekiya, M. Abe, T. Haino, *Chem. Asian. J.*, **2019**, *14*, 1786-1791.

[3] *Gaussian 09*, Revision D.01, M. J. Frisch, G. W. Trucks, H. B. Schlegel, G. E. Scuseria, M. A. Robb, J. R. Cheeseman, G. Scalmani, V. Barone, B. Mennucci, G. A. Petersson, H. Nakatsuji, M. Caricato, X. Li, H. P. Hratchian, A. F. Izmaylov, J. Bloino, G. Zheng, J. L. Sonnenberg, M. Hada, M. Ehara, K. Toyota, R. Fukuda, J. Hasegawa, M. Ishida, T. Nakajima, Y. Honda, O. Kitao, H. Nakai, T. Vreven, J. A. Montgomery, Jr., J. E. Peralta, F. Ogliaro, M. Bearpark, J. J. Heyd, E. Brothers, K. N. Kudin, V. N. Staroverov, T. Keith, R. Kobayashi, J. Normand, K. Raghavachari, A. Rendell, J. C. Burant, S. S. Iyengar, J. Tomasi, M. Cossi, N. Rega, J. M. Millam, M. Klene, J. E. Knox, J. B. Cross, V. Bakken, C. Adamo, J. Jaramillo, R. Gomperts, R. E. Stratmann, O. Yazyev, A. J. Austin, R. Cammi, C. Pomelli, J. W. Ochterski, R. L. Martin, K. Morokuma, V. G. Zakrzewski, G. A. Voth, P. Salvador, J. J. Dannenberg, S. Dapprich, A. D. Daniels, O. Farkas, J. B. Foresman, J. V. Ortiz, J. Cioslowski, and D. J. Fox, Gaussian, Inc., Wallingford CT, 2013.
